# Nanomedicine-Based Neuroprotective Strategies in Patient Specific-iPSC and Personalized Medicine

**DOI:** 10.3390/ijms15033904

**Published:** 2014-03-04

**Authors:** Shih-Fan Jang, Wei-Hsiu Liu, Wen-Shin Song, Kuan-Lin Chiang, Hsin-I Ma, Chung-Lan Kao, Ming-Teh Chen

**Affiliations:** 1 School of Medicine, National Yang-Ming University, No. 155, Sec. 2, Linong Street, Taipei 11221, Taiwan; E-Mails: lionel.lu@gmail.com (W.-S.S.); leahchiang1991@gmail.com (K.-L.C.); lkao@vghtpe.gov.tw (C.-L.K.); 2 Department of Neurological Surgery, Tri-Service General Hospital and National Defense Medical Center, No. 325, Sec. 2, Cheng-Kung Road, Taipei 11490, Taiwan; E-Mails: liubear0812bear@yahoo.com.tw (W.-H.L.); uf004693@mail2000.com.tw (H.-I.M.); 3 Graduate Institute of Medical Sciences, National Defense Medical Center, No.161, Sec. 6, Minquan E. Rd., Neihu Dist., Taipei City 114, Taiwan; 4 Department of Neurosurgery, Cheng-Hsin General Hospital, No.45, Zhenhua St., Beitou Dist., Taipei City 112, Taiwan; 5 Department of Medical Research & Education, Taipei Veterans General Hospital, No. 201, Sec. 2, Shih-Pai Road, Taipei 11217, Taiwan; 6 Department of Physical Medicine and Rehabilitation, Taipei Veterans General Hospital, No. 201, Sec. 2, Shih-Pai Road, Taipei 11217, Taiwan; 7 Department of Neurosurgery, Neurological Institute, Taipei Veterans General Hospital, No. 201, Sec. 2, Shih-Pai Road, Taipei 11217, Taiwan

**Keywords:** induced pluripotent stem cells, personalized medicine, neurodegenerative, nanoparticles

## Abstract

In recent decades, nanotechnology has attracted major interests in view of drug delivery systems and therapies against diseases, such as cancer, neurodegenerative diseases, and many others. Nanotechnology provides the opportunity for nanoscale particles or molecules (so called “Nanomedicine”) to be delivered to the targeted sites, thereby, reducing toxicity (or side effects) and improving drug bioavailability. Nowadays, a great deal of nano-structured particles/vehicles has been discovered, including polymeric nanoparticles, lipid-based nanoparticles, and mesoporous silica nanoparticles. Nanomedical utilizations have already been well developed in many different aspects, including disease treatment, diagnostic, medical devices designing, and visualization (*i.e.*, cell trafficking). However, while quite a few successful progressions on chemotherapy using nanotechnology have been developed, the implementations of nanoparticles on stem cell research are still sparsely populated. Stem cell applications and therapies are being considered to offer an outstanding potential in the treatment for numbers of maladies. Human induced pluripotent stem cells (iPSCs) are adult cells that have been genetically reprogrammed to an embryonic stem cell-like state. Although the exact mechanisms underlying are still unclear, iPSCs are already being considered as useful tools for drug development/screening and modeling of diseases. Recently, personalized medicines have drawn great attentions in biological and pharmaceutical studies. Generally speaking, personalized medicine is a therapeutic model that offers a customized healthcare/cure being tailored to a specific patient based on his own genetic information. Consequently, the combination of nanomedicine and iPSCs could actually be the potent arms for remedies in transplantation medicine and personalized medicine. This review will focus on current use of nanoparticles on therapeutical applications, nanomedicine-based neuroprotective manipulations in patient specific-iPSCs and personalized medicine.

## 1. Introduction

In the past few decades, nanoparticles (NPs) have been widely investigated in a verity of research, such as drug delivery vehicles [1], targeting delivery [2], and imaging [3]. NPs are of several unique physical properties based on their nanostructure. For example, the high surface area of NPs is capable of carrying therapeutic agents or targeting moieties for drug delivery. The inert nature of NPs makes them an ideal carrier for various drugs or molecules. In addition, NPs are able to entrap the drugs and protect them from degradation while in circulation, consequently, reducing the amount of drug required to administrate.

Neurons are particularly susceptible to hypoxia since they have an obligative aerobic glycolytic metabolism. Therefore, brain cells are extremely sensitive to oxygen deprivation and would die within five minutes after oxygen supply has been cut off. Drug delivery to the central nervous system (CNS) is very difficult due to its anatomical and physiological complexities. The CNS is protected by the blood-brain-barrier (BBB), which is composed of tightly joined capillary endothelial cells that plays a role in limiting the access of large molecules to the brain [4]. By virtue of the development in brain targeting delivery systems, some drugs encapsulated by NPs that were conjugated with BBB targeting ligands can be transported directly into the brain [5].

Over the past decade, the use of stem cells has opened up a new horizon in clinical treatment. Due to the capability of differentiating into multiple lineages of somatic cells, stem cells can be a strong tactic in regeneration or, replacement of damaged cells/tissues. Recent novel techniques have demonstrated induced pluripotent stem cells (iPSCs) could be generated from mouse embryonic fibroblasts (MEFs) and from human fibroblasts using retroviral transfection of four transcription factors Oct-4, Sox2, c-Myc, and Klf4 [6–9].

This review focuses on current applications of NPs in drug delivery and the progress of recent research on patient-specific iPSCs. Meanwhile, we point out the potential of nanotechnology as it specifically relates to developing novel techniques on cell reprogramming, which in combination, would most likely provide a solution of personalized medicine against neurodegeneration, such as Alzheimer’s disease, Parkinson’s disease and Huntington’s disease.

## 2. Nanomedicines

Nanotechnology has become a fast growing field with potential applications in medical research and drug therapy. In addition, modern innovations and evolutions in nanotechnology have revolutionized cancer therapeutics [10]. NPs are of unique physical and chemical properties due to the nanosize effect. NPs of diameter in the range between 10–200 nm are able to interact with biological systems at the molecular level [11]. Numerous studies have shown the capability of using NPs as the carrier/vehicle for cancer therapies. NPs have the ability to encapsulate, or to form a complex with drug molecules. In some studies, NPs have been shown to deliver drugs to the cancerous cell without attacking the normal cells [12]. In addition, with the combination of fluorescent [13] or radiolabeling material, NPs have made a great contribution in the research of tracking/imaging of drug delivery in both *in vitro* and *in vivo* models. What is more, nanoencapsulation of drug/macromolecules increases drug efficacy, specificity, tolerability, and enhances the therapeutic level of the corresponding drugs [14]. These NPs are able to protect premature degradation of the incorporated drugs while interacting with the biological environment. Moreover, due to their nanosize structure, these NPs can facilitate absorption, prolong retention time, and, help cellular penetration of the drug that is encapsulated inside [15]. Therefore, nanoencapsulated formulations are generally regarded as of higher efficacy and lower toxicity due to the lower amount of drug required for administration [16]. Many nanoparticulated drug delivery systems have been created, including liposomes, solid lipid nanoparticles (or nanostructure lipid carriers), polymeric nanoparticles (*i.e.*, polylactides and poly (lactic-*co*-glycolic)), dendrimer, and metallic nanoparticles. A number of studies have demonstrated that the accumulation of nanostructured carriers in the tumor microenvironment through the so-called, “enhanced permeability retention (EPR)” effect is an advantage of nanoparticulated drugs [17]. Although hundreds of studies have been done on nanoparticulated drug delivery systems, there are only about 20 nanoparticulated formulations of therapeutic effect that have received FDA approval for clinical use [18]. Here, we introduce several nanoparticulated carriers, summarized in Table 1, which is currently widely focused on.

### 2.1. Solid Lipid Nanoparticles (SLNs)

Due to the high biocompatibility and low toxicity, lipid-base nanoparticulated vesicles have been extensively studies in the past few decades. The developments of several well-known lipid-originated delivery platforms have tremendously sped up the discoveries of numerous medical/health applications. These carriers, such as solid lipid nanoparticles, nanoparticulated lipid carriers, and liposomes, are able to entrap the therapeutic agents (*i.e.*, drug, proteins or DNA) inside their pockets and provide protection against the physiological environment, hence increase the bioavailability of the drug, while reduce the possible side-effects of the carrying drug. SLNs have recently been proposed for oral [19,20], topical [21] and parenteral [22] administration. SLNs consist of solid lipids in nanosized range (50 to 500 nm), which are generally dispersed in an aqueous medium. SLNs have been proposed to be a feasible carrier for peptide or protein delivery, because they have the benefits of both liquid-based colloidal systems (such as liposomes and emulsions) and solid formulations [23].

Since SLNs are composed of biocompatible and biodegradable natural or synthetic lipids, SLNs are highly preferable for delivery of biopharmaceuticals. Moreover, large scaled production of SLNs can be performed in a cost-effective and relatively simple way (such as high pressure homogenization). For the location of drugs in the particle, SLNs can be separated into three models, (i) the homogeneous matrix model; (ii) the drug-enriched shell model; and (iii) the drug-enriched core model [24,25]. The morphological differences of the three models depend mainly on the composition of the formulation, which includes the chemical property of the pharmaceutical ingredients, lipids, and surfactants, as well as the manufacturing method. In the homogeneous matrix model, drugs are dispersed in the lipid matrix via the cold high-pressure homogenization process in which the bulk lipid contains the dissolved drugs in molecularly dispersed form. Homogeneous matrix models can also be achieved by using hot high-pressure homogenization, provided that no phase separation of the lipid and drugs occurs. In the homogeneous matrix model, the drugs are entrapped in the lipid matrix, which allows prolonged drug release from SLN. When phase separation occurs during the cooling process of the liquid oil droplet to the SLN in hot high-pressure homogenization, a drug-free lipid core will form if the lipid precipitates first. Lastly, the drugs can be cooled and crystallized as a drug-enriched shell. In this case, drug should be released very rapidly from the SLNs [25]. A drug-enriched core is formed with the opposite approach. The drugs precipitate and crystallize first and form a core, while the lipids crystallize later to form a shell of the SLNs. In this model, drugs are released from the SLNs in a membrane-controlled manner, which follows the Fick’s Law of diffusion [21]. Because SLNs are composed of naturally available lipids of lower melting points (fatty acids, triglycerides, and their derivatives), SLNs can be generated without (or at a minimum amount of) the use of harsh solvents, which makes SLNs a safer protein/micromole delivery platform for different routes of administration, such as, intravenous, oral and topical delivery.

### 2.2. Liposomes

Liposomes are uni-lamellar or multi-lamellar spherical vesicles primarily comprised of phospholipids (from plant or animal source) and were first revealed by A.D. Bangham at Babraham, Cambridge in 1961 [26]. These lipid-based microparticles and nanoparticles are usually manufactured from naturally derived, biocompatible phospholipids; therefore, these vesicles are considered the least toxic, compared to the other polymeric nanoparticles. Due to the physical structure of polar core and lipophilic bilayer, liposomes are capable of entrapping both hydrophilic and hydrophobic drugs and macromolecules (*i.e.*, peptides and proteins). Liposomal encapsulation works as a shield for the loaded drugs against degradation in physiological environment and thus, increases the stability of the drug while in circulation.

There have been numerous studies investigating the controlled-release manner of liposomal drug delivery systems. For example, a single subconjunctival injection of latanoprost encapsulated EGG-phosphatidylcholine (EGG-PC) liposomes (averaging about 110 nm in diameter) was able to sustainably release the drug and maintained a low intraocular pressure (IOP) in rabbit eyes for up to 90 days [27]. Here liposomes acted as a depot system in the subconjunctival space. The size of the EGG-PC liposomes was able to prolong the retention time at subconjunctival space with limited systemic clearance. In addition, liposomal encapsulation might also facilitate the delivery of the drug through various anatomical structures of the eye (such as conjunctiva and sclera) and more efficiently arrive at the desired sites (ciliary body), which ultimately, leads to the improved bioavailability.

Several modifications have been performed on liposomal applications. For example, PEGylated liposomes (stealth liposomes) have been explored and displayed an enhanced permeability retention (EPR) effects in cancerous tissues, as well as a reduction in systemic side effects of anticancer agent [28]. PEGylated, drug-loaded liposomes could be further surface-modified for targeted delivery against tumor tissues. For example, daunorubicin, an injectable drug widely used in chemotherapy, was incorporated on the surface of modified liposomes with encapsulated quinacrine for treating and preventing the recurrence of breast cancer arising from the cancer stem cells [29].

However, PEGylated modification of liposomes is actually a dilemma. This is because, although PEGylation have been demonstrated to stabilize drug-loaded liposomes and prolong their blood-circulation time [30], the hindered cell-uptake and incomplete drug release of PEGylated liposomes could still result in low drug bioavailability at the cancerous site, which leads to inadequate therapeutic effect [31]. Recently, cross-linked, multilamellar liposomes (CML) for controlled delivery of anticancer agents have been exploited to improve sustainable drug release kinetics and enhanced the stability of the liposomes [32]. This enhanced vesicle stability contributed to higher doxorubicin (Dox) bioavailability and improved *in vivo* therapeutic activity against tumors. Notably, CML-Dox exhibited significant inhibition of B16 tumor (one of the most aggressive types of tumors) growth, compared to that treated with the conventional liposomes. In addition, the enhanced therapeutic efficacy of CMLs resulted from the augmented accumulation of drugs was shown at tumor sites, while a much lower accumulation of CMLs in heart and spleen was found. These results implied the improvement in effectiveness and safety of CML-encapsulated drugs by minimizing the unwanted side effects.

### 2.3. Polylactides and Poly (Lactic-*co*-Glycolic) Acid Nanoparticles

Polyesters such as polylactic-*co*-glycolic acid (PLGA) are widely used polymers for the delivery system by nano-formulation. PLGA is a copolymer of lactic acid and glycolic acid. After uptake in the human body, PLGA undergoes hydrolysis and is degraded into biodegradable metabolite monomers (lactic acid and glycolic acid). Therefore, PLGA is of minimal systemic toxicity, and considered safe for different routes of administration, such as oral, topical, and intravenous delivery. The degradation rate of PLGA is dependent on the molar ratio of lactic acid and glycolic acid, molecular weight of the polymer, and the glass transition temperature of the polymer [33]. PLGA is one of the most successfully used safe biodegradable polymers and approved by the US FDA and European Medicine Agency (EMA) in various drug delivery systems in humans [34]. PLGA has a well-described formulation and methods of production adapted to hydrophilic or hydrophobic small molecules or macromolecules [35]. Additionally, it protects the drug molecule from degradation and exhibits sustained release [36]. Most importantly, PLGA-based NPs are able to target particles to specific organs or cells [37,38], and have been widely used as drug delivery systems for the treatment of different pathologies. A large number of studies have demonstrated applications of PLGA-based delivery nanosystems in vaccination [39,40], cancer treatment [41,42], inflammation [43], and regenerative medicine [44]. PLGA-based NPs also provide promising ocular delivery models for sustained drug release in the treatment of ocular inflammation [45]. In addition, PLGA NPs were able to enhance ocular permeability of inflamed corneal surface without toxicity. Also, several studies have reported evaluations of curcumin loaded PLGA NPs (Cu-NPs) [46,47], such as improved water solubility, higher release rate in the intestinal fluid, enhanced absorption by improved permeability, and increased residence time in the intestinal cavity, which may be associated with the improved oral bioavailability of curcumin.

## 3. Stem Cell Biology and the Utility of Cell Therapy

Nanotechnology has provided the possibility of new therapeutic opportunities for active pharmaceutical ingredients (APIs) that cannot be used effectively in conventional formulations due to poor bioavailability or drug instability. Based on the nanoscaled properties (*i.e.*, high surface area for API carrying, specific targeting, and lower toxicity), NPs have been greatly investigated as so-called “Nanomedicines” for many decades, especially in cancer therapies. For example, the US Food and Drug Administration (FDA) approved an anti-metastatic breast cancer formulation, AbraxaneTM, albumin-paclitaxel (TaxolTM) NPs in 2005. However, activity in nanotechnology innovation in stem cell biology and cell reprogramming remains low. In the next few sections, we focus on the most up-to-date applications in stem cell biology, and the breakthrough point of nanotechnology in iPSCs research and utility.

### 3.1. Adult Stem Cells

Based on their abilities of differentiation, stem cells can be categorized into three types. The first type of stem cells is totipotent stem cells, which can be implanted in the uterus of a living animal and gives rise to a full organism. The second type of stem cells is iPSCs; including embryonic stem (ES) cells and iPSCs. They can give rise to every cell of an organism except extraembryonic tissues. This limitation restricts iPSCs from developing into a full organism. The third type of stem cells is multipotent stem cells. They are adult stem cells, which only generate specific lineages of cells. The existence of various types of adult stem cells has been reported, including hematopoietic stem cells, mesenchymal stem cells, neural stem cells, neural crest stem cells, and so on. A brief review of adult stem cell research is provided below.

#### 3.1.1. Hematopoietic Stem Cells

Adult stem cells are required for a lifelong sustenance of matured cell replacement and hold great promise for future therapeutic applications. Hematopoietic and endothelial cells can be developed from a common postnatal progenitor, such as, the hemangioblast [48,49] in both *in vitro* and *in vivo* models. In clinical stem cell transplantation for the treatment of leukemia [50], hematopoietic stem cells (HSCs) are currently being used. HSCs are also used in the treatment of many non-hematological disorders, such as autoimmune diseases and metabolism disorders [50]. Advanced characterization of hemangioblasts will be important for a good understanding of the molecular events involved in stem cell properties and for using this cell population for clinical applications. In addition, in order to further exploit their potential in therapeutic applications, a better understanding of HSCs can help the *ex vivo* expansion of the HSCs or the *in vivo* control of their differentiation directions.

#### 3.1.2. Bone Marrow-Derived Stromal Stem Cells

Bone marrow is a complex tissue containing stem cells for hematopoietic cells, and stem cells that are precursors of non-hematopoietic tissues. The precursors of non-hematopoietic tissues have the ability of becoming one of a number of phenotypes, which are capable of self-renewal, but without differentiation. These non-hematopoietic tissues can serve as a feeder layer that supports hematopoietic stem cell growth. They were initially called plastic-adherent cells or colony-forming-unit fibroblasts, and subsequently renamed either as marrow stromal cells or mesenchymal stem cells (MSCs). Extensive *in vitro* and *in vivo* experimentation has defined conditions for the isolation, propagation, and differentiation of MSCs.

#### 3.1.3. Neural Stem Cells

Neural stem cells (NSCs), derived from the hippocampus and other germinal centers of the brain, have been isolated and defined as cells capable of self-renewal and multilineage differentiation [51]. NSCs also have the utilizing potential to develop the transplantation strategies. In addition, NSCs can also be used to screen the candidate agents for neurogenesis in neurodegenerative diseases [52]. In the adult brain, the location of NSCs is primarily in the subgranular zone (SGZ) of the hippocampal dentate gyrus and the subventricular zone (SVZ) of the lateral ventricle. In general, the quiescent or dormant NSCs might be present and can be derived from multiple areas of the adult brain [53–55]. The SVZ and SGZ niches have common cellular niche components which include vascular cells, ependymal cells, astroglia, NSC progeny and mature neurons, and common extracellular niche signals, including Sonic Hedgehog, Wnt, bone morphogenic protein antagonists, leukemia inhibitory factor, membrane-associated Notch signaling transforming growth factor-alpha, fibroblast growth factors, extracellular matrix and neurotrophin. These cellular and extracellular components regulate the behaviors of NSCs in a region-specific manner [54].

### 3.2. Embryonic Stem Cells

Embryonic stem cells (ES cells) are iPSCs derived from the inner cell mass of mammalian blastocysts. They have abilities to proliferate indefinitely under appropriate *in vitro* culture systems and to differentiate into any cell type of all three germ layers [56–58]. Since the successful isolation of human ES in 1998, ES cells have been regarded as a powerful platform/tool for developmental studies, tissue repair engineering, diseases treatment, drug screening, and regenerative medicine. However, two main limitations have impeded the application of ES cell-based therapy: The ethical dilemma regarding the human embryo donation/destruction, and incompatibility with the immune system of patients.

### 3.3. Cell Reprogramming and iPSCs

Scientists have been devoted to developing a variety of reprogramming techniques to reverse somatic cells into a stem cell-like state [59] to circumvent the deficiencies. In 2006, Takahashi and Yamanaka [60] made a landmark discovery: Reprogramming of somatic cells back to iPSCs through retroviral transduction of four pluripotency-associated transcription factors, Oct3/4, Sox2, c-Myc, and Klf4. Most importantly, these iPSCs possess morphological and molecular features that resemble those of ES cells and give rise to teratoma and germline-competent chimeras after injection into blastocysts. iPSCs closely resemble ES cells in terms of self-renewal capacity, epigenetic profile (such as DNA methylation and miRNAs), global gene expression, and developmental potential. Moreover, the iPSCs technique promises unprecedented opportunities for the advancement of disease modeling and for studying the fundamental biology underlying epigenetic reprogramming, drug screening and personalized-specific cell therapy [61]. However, several critical disadvantages of the traditional iPSCs technique hinder many practical applications of the technology. These disadvantages include: Delivery of vectors could be very harmful, and the reprogramming process may be of low efficiency and with slow kinetics [62,63]. In addition, the above-mentioned characteristics of reprogramming process may also present some “hidden” risks for iPSCs. For example, inappropriately induced epigenetic changes, epigenetic memory, and accumulation/selection of other subtle epigenetic and genetic abnormalities found during the process of reprogramming.

To overcome such drawbacks of viral transduction in cell reprogramming, a feasible approach using protein, mRNA, microRNA or small molecules that can enhance reprogramming efficiency, fasten kinetics, and functionally replace exogenous reprogramming transcription factors has been studied in recent years [64]. Cao and co-workers used calcium phosphate nanoparticles (CPNPs) as a vehicle for the generation of virus-free iPSCs from human umbilical cord mesenchymal stem cells (HUMSCs) by co-delivery of the four plasmids (Oct4, Sox2, Klf4, and c-Myc). Unlike traditional viral induction with low reprograming efficiency (RE), a remarkably enhanced RE was achieved (about 0.049%) using these CPNPs. The generated iPSCs show positive expression of pluripotency markers (OCT4, SSEA-3, SSEA-4, NANOG, and TRA-1-81) and are able to differentiate into all three germ layers *in vitro*. Furthermore, after subcutaneous injection of these iPSCs into immune-compromised mice, the formation of teratomas containing a variety of tissues from all three germ layers was also validated [65]. Recently, studies have pointed out that certain microRNAs are essential supporters of genes that regulate pluripotency and are highly expressed in ES cells and vital to generate iPSCs [66]. Sohn *et al.* demonstrated a successful activation of pluripotency-associated genes in mouse bone marrow (BM) mononuclear cells using ES cell-specific microRNAs encapsulated in the acid sensitive polyketal (PK3-miRNAs) nanoparticles [66]. After eight days of treatment, these PK3-miRNAs particles induced pluripotent gene and protein expression in cultured mouse BM-derived mononuclear cells (MNCs). Pluripotency markers, Oct4, Sox2, and Nanog were also founded in the isolated cell colonies. In addition, colonies transferred to feeder layers also stained positive for pluripotency markers including SSEA-1. These findings implied that a NP-based delivery vehicle could generate various reprogrammed cells without permanent genetic manipulation in an efficient manner.

## 4. Patient-Specific iPSCs and Neurodegeneration-iPSCs

Neurological disorders have been studied for decades, yet the underlying mechanisms and the treatments are still unclear. Part of the reason is that the tissue samples from patients’ brains are often difficult to obtain. There are also obstacles to establish animal models, including incapability of recapitulating all pathophysiological characteristics of a specific disorder [67]. Nevertheless, the emergence of iPSCs is a landmark in recent neurological research and drug development.

iPSCs technology was first demonstrated by Takahashi and Yamanaka in 2006. They induced pluripotency to adult mouse fibroblasts by overexpression of four genes expressed in ES cells: Oct3/4, Sox2, Klf4, and cMyc [60]. This novel method is remarkable in that it is completely independent of the access to ES cells, and allows the researchers to avoid ethical quandary in terminating human embryonic development. Another advantage of applying iPSCs as a research tool is based on the ability of iPSCs to reserve epigenetic features of individual patients [68]. On the other hand, it is difficult to set up both animal and cellular models in the earlier stage of neuroscience research. For example, in terms of Huntington’s disease, a mouse model almost matches progressive outcomes but fails to fully coincide with genetic and pathological features on human beings [69]. Immortalized rat cells with mutant STHdhQ111 were introduced to overcome the limitation of animal models. However, the above-mentioned rodent genetic heritage, and abnormal cell physiology caused by the immortalization process leads to impracticable application of the drug-testing platform. Besides, overexpression of known disease genes, which give rise to aberrant proteins result in the failure of informing true disease process in human patients [70]. Application of iPSCs can therefore be an efficient way to solve the above-mentioned problems. Since iPSCs can be obtained directly from a patient, the subsequent drug screening or therapeutic treatment results can actually provide existing pattern of mutations, duration, and severity of the specific disease of that patient. Therefore, this personalized screening/treatment can generate a more reliable platform compared with modeled disease phenotype, and enable researchers to monitor actual disease progression and reaction to new drug treatments on a patient’s own manner [67].

Degenerative neurological disorders have attracted an increasing prevalence nowadays. They share similar pathological features, which includes, delayed onset, specific neuronal damage, and protein dysfunction [71]. Oxidative stress is considered as a risk factor in the incidence and progression of cognitive declines that occur during normal cerebral aging and dementia and plays a critical role in many neurodegenerative disorders, such as Alzheimer’s disease and Parkinson’s disease [72]. Hence, the development of human iPSCs may actually provide novel utility towards establishing disease phenotype for the following clinical studies and drug screening.

### 4.1. Huntington’s Disease

Huntington’s disease (HD) is a debilitating neurological disorder, which characterizes cognitive, motor, and emotional alteration. It is an autosomal-dominant genetic disease caused by expanded CAG repeats in the first exon of the Huntingtin (HTT) gene [71,73]. The expression of over 40 CAGs will consistently cause HD, and the earlier disease onset is predicted when the CAG length expands longer [74,75]. The expanded region causes huntingtin protein to aggregate in the nucleus of certain neurons, which leads to brain cell death, especially striatal medium spiny neurons that express dopamine- and cAMP-regulated phosphoprotein [73]. Even though the genetic mutation of HD is clear, there is no treatment to cure or slow down the pathological progression.

Recently human iPSCs were successfully established into cell lines from a HD patient with a 72-repeat CAG tract [76]. These patient-specific-iPSCs were later reprogrammed into a neuronal state, which may serve as a suitable disease phenotype due to the same expression of the expanded CAG feature, along with the characteristic elevating caspase-3/7 activity of the original iPS cell line [77]. However, there is not enough evidence to support study of HD-iPSCs phenotypic differences. The HD iPSCs consortium, which gathers the effort from eight international research groups, has generated a large panel of iPS cell lines from various patients [73,78]. They uncovered numerous HD phenotypes with observation of either CAG-length dependent or independent expression, including cell toxicity and the loss of brain-derived neurotrophic factors (BDNF) [73]. The utility of this HD-iPSCs model, with characterization of multiple clones and diverse repeat lengths, will therefore, help elucidate HD pathological mechanism and progression, which ultimately, contribute to the development of following drug screening or novel therapeutic treatments [73].

### 4.2. Parkinson’s Disease

Parkinson’s disease (PD) is an idiopathic ailment that involves smooth motor inability and cognitive dysfunction with aggrandizing prevalence along with age [79,80]. The exacerbation of the disease progress involves degeneration of dopaminergic neurons within the substantia nigra [81]. Although for the last decade, the associated pathological genes (including *PARK7*, *LRRK2*, *PINK1*, *SNCA*, *UCHL1*, *GBA*, and *SNCAIP*) have been studied [82], cases reported remain sporadic, and are more likely a result of a complex combination between genetic and environmental factors [83]. Recently, the derivation of human iPSCs from PD patients provided an insight into the underlying pathophysiology and thus may solve the difficulty of the dearth of reliable experimental models that recapitulate essential disease features [84].

Soldner *et al.* were the first to generate human iPSCs from five idiopathic PD patients [84]. Interestingly, they verified the efficiency of human iPSCs generation through reprogramming-factor-free protocol, which excises the transgenes by Cre-recombinase and is capable to differentiate to functional dopaminergic neurons. These factor-free human iPSCs not only maintain pluripotency, but also greatly reduce the risk of oncogenesis due to re-expression of transcriptional factors [84,85]. In the follow-up study, Soldner *et al.* generated a defined genetic background by using zinc finger nucleases (ZFNs) to exclusively edit or correct a single base pair (e.g., A53T or E46K), while reserving residual genetic background [80,86]. The production of isogenic control cell lines can provide a panel to identify drug efficiency on different mutation samples or to screen genetic and small molecule disease modifiers on a large-scale basis, which represents great progress not only for basic bimolecular research but also for human-iPSCs-based cell replacement therapy [86].

### 4.3. Alzheimer’s Disease

Alzheimer’s disease (AD) is a fatal neurodegenerative disease without complete understanding or effective cure so far. Although most AD patients are sporadic with non-Mendelian genetic contribution, research has elucidated that extracellular amyloid plaques consisted of Aβ fragments of amyloid precursor protein (APP) and intraneuronal neurofibrillary tau tangles are dominant in pathogenesis [87–89]. Recent analyses of familial Alzheimer’s disease derived from human iPSCs further implicate that genetic mutation is possibly related to mechanisms of the disease.

In a prior study, human-induced neuronal cells are generated from skin fibroblasts of nine AD patients, which consists of non-demented and familial forms. Cells derived from familial AD patients, which express presenilin (PSEN) gene mutations display relatively higher Aβ42/Aβ40 ratios to those derived from unaffected individuals, which is a typical feature in the familial form yet with an amplified expression [88,89]. Although this well-characterized phenotype can provide a comparably useful platform for further investigation on pathological mechanisms and drug therapeutic effectiveness, just two disease forms cannot be representative of the true pathogenesis of the entire disease population. An advanced investigation on other sporadic disease forms is therefore conducted. Compared with unaffected control individuals, neurons derived from both familial and sporadic patients exhibit significantly increasing pathological markers of Alzheimer’s disease, which are amyoid-β (1–40), phospho-tau (Thr231) and active glycogen synthase kinase (aGSK-3β) and RAB5-positive early endosomes, respectively [89]. This suggests that sporadic Alzheimer’s disease patients exhibit similar phenotypes to familial AD samples, though maybe not always.

The development of human iPSCs technology in neurodegenerative disorders has been booming and prosperous; however, there are barriers while transferring iPSCs to clinical application. First, occurrence of teratoma after autologous transplant in animal models has increased the awareness of the clinical application of iPSCs [90]. Even though the issue can be fixed by epigenetic modification, high cost, time-consuming production and complicated analyses make the treatments to cure acute or life-threatening diseases impractical [91,92]. To avoid the risk of developing cancer-like resultant cells, the rapid iPSCs reprogramming approach through the usage of small molecules has evolved, which is of significant efficiency in producing iPSCs, and capable of reducing unwilling oncogenesis occurrence [93]. Recent research on induction of pluripotency using combinations of a single gene mutation and small molecules was accomplished in mouse somatic cells [94]. This has shed a promising light on the optimization of cellular reprogramming protocols involving small molecules alone. Nevertheless, although small molecules can increase reprogramming efficiency, the production rate remains lower that that by using other protocols. Besides, selection of small molecules for reprogramming various cells is also a challenge to be overcome [92]. Therefore, it is still a distant option for iPSCs to be use in clinical applications. Optimal cell sources and careful validation are necessary for minimizing safety concerns, which can therefore allow the possibility of novel treatment in the future.

## 5. Applications Patient-Specific iPSCs and Drug Screening Using Nanoparticles (Personalized Medicine)

Current progress in induced iPSCs technology has successfully generated human iPSCs by forced expression of transcription factors in somatic cells, thus providing new opportunities for regenerative medicine and *in vitro* disease modeling [95]. Several attempts have been made to generate iPSCs from patients with various diseases [96]. For example, patient-specific iPSCs have been differentiated into neural crest precursors, motor neurons, and mature hepatocytes [97]. This experimental data demonstrated that human iPSCs could be used to model the specific pathogenesis of a genetically inherited disease, to screen candidate drugs, and to facilitate cell replacement therapy. Retinal pigment epithelium (RPE), a monolayer of cells located beneath the neurosensory retina, plays essential roles in retinal homeostasis, including the formation of the blood-retinal barrier, visual pigment regeneration, and phagocytosis of shed photoreceptor discs [98]. Although it has been accepted that the RPE is the major pathogenic target of Best Disease (BD), obtaining a sufficient number of RPE cells and photoreceptors from suitable donors for drug screening and disease modeling has remained an obstacle. Recently, Osakada *et al.* reported that human iPSCs are capable of differentiation into differentiated retinal progenitors, RPE cells, and photoreceptors [99]. Singh *et al.* recently generated BD-patient-specific iPSCs (BD-iPSCs) and differentiated these cells into RPE-like cells [100]. Unfortunately, whether patient-specific iPSCs-derived RPE-like cells can be adopted as an expandable platform for high-throughput drug screening, remains unclear.

Personalized medicine is an emerging medical practice that proposes the customization of healthcare for the prevention, diagnosis, and treatment of disease. Personalized medicine would be tailored to the individual patient based on the patient’s unique genetic profile [101]. Recently, numerous studies have focused on human iPSCs due to their ability to propagate infinitely while maintaining the ability to differentiate into many different cell types of the human body [102]. The high consistency, purity, and expandability of human iPSCs facilitate drug screening, toxicity testing, and the development of personalized medicine for the treatment of degenerative diseases [95]. These patient-specific iPSCs and their differentiated progenies have provided models for particular individualized disease phenotypes that are useful in understanding mechanisms in the diseases and investigating the pathogenesis of disease-causing mutations [103]. The integration of nanomaterials and biology has influenced modern nanomedicine, especially towards personalized medicine [104].

Although NP-based drug delivery systems have been greatly investigated, especially in cancer therapies, not much emphasis has been placed on the application of NPs in patient-specific iPSCs, or patient-specific drug screening. However, based on the nanoscaled structure and tremendously large surface area, we strongly believe that NPs can be a powerful weapon in both reprogramming of disease-specific iPSCs and the following drug screening process performed on the obtained disease-specific iPSCs. For example, mesoporous silica nanoparticles (MSNs) have superior biocompatibility and their high surface area could contribute to high drug loading, which enables MSNs as an ideal platform for drug delivery and drug screening. Their excellent biocompatibility makes MSNs better multifunctional nanomaterials for biomedical niche applications. Additionally, it has been demonstrated that MSNs with various surface charges could be efficiently internalized by iPSCs without causing cytotoxicity [105]. MSNs also serve as an ideal vector for non-viral stem cell labeling, gene delivery, and as a potential drug delivery platform for inducing specific differentiation and cell-oriented therapeutics. Our recent study demonstrated that 100 nm FITC-conjugated MSNs (FMSNs) can efficiently enter 3T3-L1 cells and human mesenchymal stem cells [105]. However, there is a gap between nanotechnology and iPSCs even till now. The manner in which NPs interact with iPSCs is still uncertain. Previously, Chen *et al.* validated that pHNF3β-FMSN (+) improved the iPSCs differentiation toward hepatocyte-like lineage with mature liver function, and double delivery of pHNF3β-FMSN (+) further improved mRNA-expression levels of liver specific genes. They also demonstrated that the MSNs with various surface charges could be efficiently internalized by iPSCs without causing cytotoxicity. In addition, the levels of reactive oxygen species and pluripotent status, including *in vitro* stemness signatures and *in vivo* teratoma formation remained unaltered. Therefore, FMSNs with multifunctional properties are a suitable delivery vehicle for biomolecule delivery and can serve as an ideal vector for stem cell labeling, and gene delivery, as well as a potential drug carrier for inducing patient-specific differentiation and the subsequent personalized therapeutics.

## 6. Conclusions

In this article, we have explored different types of NPs serving as therapeutic agents of various aspects of treatments. Based on the unique physical properties, NPs are supposed to be qualified to penetrate the BBB and deliver the encapsulated/carried drugs to the desired site in the brain. Therefore, NPs can be a promising tool to treat currently prevailing neurodegenerative diseases. On the other hand, explorations on patient-specific iPSCs open up a new strategy on personalized medicines. Together, nanotechnology and patient-specific iPSCs have become the enabling technologies for personalized medicine, in which the genetic information could be used to predict disease development, progression and clinical outcomes. However, we have not witnessed many successful inventions in NP-based formulations for the treatment or prevention of neurodegenerative diseases, such as AD and PD so far. Nevertheless, the future prospects for this technology remains bright; further progress will be required to convert the concepts of drug-encapsulated NP technology and iPSCs into practical innovation as the new generation of personalized medicine.

## Figures and Tables

**Table 1.** Summary: Recent nanoparticulated drug delivery systems.

Type of Nanoparticles	Inventors	Year	Source	Active ingredients	Advantages	Disadvantages
Solid Lipid Nanoparticles (Solvent evaporation)	Prabhu, S. *et al.*	2005	International Journal of Pharmaceutics	Piroxicam	-Improved permeation. -Good stability over 6 months.	-Declined dissolution rate after 6 months stability test. -Crystallization of solid dispersions upon storage.
Solid Lipid Nanoparticles (W/O/W emulsification)	Gallarate, M. *et al.*	2009	Journal of Microencapsulation	Insulin	-Successful encapsulation of hydrophilic API. -Protection of encapsulated peptide API. -Less harsh solvent required during the manufacturing process.	-Low encapsulation efficiency (about 40%).
Liposomes (Film hydration)	Natarajan, J.V. *et al.*	2012	International Journal of Nanomedicine	Latanoprost	-High loading efficiency (94% ± 5%). -High drug/lipid mole ratio (0.181). -Stable for at least 6 months on storage. -Sustained release (60% in 14 days, *in vitro*). -Better sustained IOP lowering.	
Liposomes (PEGylated liposomes)	Lin, Y.Y. *et al.*	2013	Plos One	NanoVNB, InNanoX, and InVNBL	-Specific tumor targeting and significantly increased tumor uptake after periodical treatment with InVNBL was evidenced. -Scintigraphic imaging of radiolabeled liposomes could provide a noninvasive screening of patients before conducting tumor treatment.	-Only efficacious at initial tumor treatment (when the tumor is relatively small or in the early stage of metastasis).
Liposomes (Film hydration)	Zhang, L. *et al.*	2012	Biomaterials	Daunorubici, quinacrine	-Successful mitochondrial targeting liposomes were developed, which were able to induce apoptosis of MCF-7 cancer stem cells. -Similar anti-breast cancer effects were observed in relapsed tumor in mice.	
Crosslinked multilamellar liposomes (Dehydration-rehydration)	Joo, K.I. *et al.*	2013	Biomaterials	Doxorubicin	-Improved stability of encapsulated drug, with better-controlled release rate. -CML-Dox showed reduced systemic toxicity and significantly improved therapeutic activity in inhibiting tumor growth.	
Polylactic-*co*-glycolic acid (PLGA) nanoparticles (Solvent evaporation)	Thote, A.J. *et al.*	2005	Drug Development and Industrial Pharmacy	Dexamethas one, dexamethasone phosphate	-Reduced burst release of drug due to surface crosslinking. -Prolonged sustained drug release achieved.	
PLA and PLGA nanoparticles (Solvent displacement)	Musumeci, T. *et al.*	2006	International Journal of Pharmaceutics	Docetaxel	-Sustained drug release (about 50% in 10 days).	-Low entrapment efficiency (less than 20%).
PLGA nanoparticles (Double emulsification method)	Gupta, S. *et al.*	2013	Drug Development and Industrial Pharmacy	Acyclovir	-Sustained release pattern. -Augmented bioavailability, increased residence time and enhanced delivery of acyclovir to the liver upon galactosylation (*in vivo*).	
PLGA nanoparticles (Nanoprecipitation, ultrasonication)	Das, M.K. *et al.*	2013	Asian Journal of Chemistry	Curcumin	-Sustained drug delivery for 2 days with no initial burst release. -Higher cytotoxicity of drug-entrapped nanoparticles over pure drug due to their efficient internalization into the tumor cells.	
